# Alpha-1 Antitrypsin for COVID-19 Treatment: Dual Role in Antiviral Infection and Anti-Inflammation

**DOI:** 10.3389/fphar.2020.615398

**Published:** 2020-12-11

**Authors:** Chengliang Yang, Shaf Keshavjee, Mingyao Liu

**Affiliations:** ^1^ Latner Thoracic Surgery Research Laboratories, Toronto General Hospital Research Institute, University Health Network, Toronto, ON, Canada; ^2^ Institute of Medical Science and Department of Surgery, Faculty of Medicine, University of Toronto, Toronto, ON, Canada

**Keywords:** alpha-1 antitrypsin (A1AT), severe acute respiratory syndrome coronavirus 2 (SAR-CoV-2), coronavirus disease 2019 (COVID- 19), acute lung injury, acute respiratory distress syndrome (ARDS), anti-inflammatory therapy, antiviral therapy

## Abstract

Many drugs have been approved for clinical trials for the treatment of COVID-19 disease, focusing on either antiviral or anti-inflammatory approaches. Combining antiviral and anti-inflammatory drugs or therapies together may be more effective. Human alpha-1 antitrypsin (A1AT) is a blood circulating glycoprotein that is best known as a protease inhibitor. It has been used to treat emphysema patients with A1AT deficiency for decades. We and others have demonstrated its role in reducing acute lung injury by inhibiting inflammation, cell death, coagulation, and neutrophil elastase activation. Recently, A1AT has been found to inhibit severe acute respiratory syndrome coronavirus 2 (SARS-CoV-2) infection by inhibiting transmembrane serine protease 2 (TMPRSS2), a protease involved in the entry of SARS-CoV-2 into host cells. This dual role of both antiviral infection and anti-inflammation makes A1AT a unique and excellent candidate for COVID-19 treatment. Three clinical trials of A1AT for COVID-19 treatment have recently been approved in several countries. It is important to determine whether A1AT can prevent the progress from moderate to severe lung injury and eventually to be used to treat COVID-19 patients with acute respiratory distress syndrome.

## Introduction

The coronavirus disease 2019 (COVID-19) pandemic has caused a surge of critically ill patients in intensive care units across the world. It has been estimated that 1.7 billion people have at least one underlying condition that puts them at increased risk of severe COVID-19 if infected (Clark et al., 2020). The underlying mechanisms of COVID-19 are severe acute respiratory syndrome coronavirus 2 (SARS-CoV-2) viral infection-induced inflammatory response, cell death, microthrombus formation, and formation of neutrophil extracellular traps (NETs) (Middleton et al., 2020). Many clinical trials, therefore, have been focused on drugs that have either antiviral infection or anti-inflammatory function. It has been suggested that a combination of anti-inflammatory drugs with direct-acting antivirals could reduce viral infectivity, viral replication, and the aberrant host inflammatory response (Stebbing et al., 2020). Drugs with a dual role in both anti-inflammation and antiviral infection could be excellent candidates for COVID-19 treatment.

## Current Available Treatments for Coronavirus disease 2019

In a large COVID-19 cohort study, 81% of patients had mild or moderate disease, 14% had severe disease, and 5% became critically ill with a life-threatening disease course. The mortality in critically ill patients with COVID-19 is approximately 49% (Wu and McGoogan, 2020). Therefore, it is urgent to have specific, effective, and safe treatments for COVID-19. Many clinical trials have been conducted to repurpose existing drugs, such as antiviral agents. To inhibit SARS-CoV-2 viral infection induced inflammatory responses, Bruton’s tyrosine kinase inhibitors and Janus kinase inhibitors have been tested. Moreover, blood-derived products, antibodies (such as interleukin-1 inhibitor and interleukin-6 inhibitors), and vaccines (Lurie et al., 2020; National Institutes of Health, 2020; World Health Organization, 2020), have also been tested clinically (Table 1). Recently, remdesivir, an antiviral drug (Beigel et al., 2020), has been approved by the United States Food and Drug Administration (United States Food and Drug Administration, 2020). However, people have raised many questions about its worth (Cohen and Kupferschmidt, 2020). New drugs should be selected and tested.

**TABLE 1:** The current drugs or therapeutics in COVID-19 clinical studies.

Type of treatment	Name	FDA approval
Antiviral agent	Chloroquine	Not yet
	Hydroxychloroquine	Not yet
	Lopinavir/ritonavir	Not yet
	Ivermectin	Not yet
	Remdesivir	Yes
Bruton’s tyrosine kinase inhibitors	Acalabrutinib	Not yet
	Ibrutinib	Not yet
	Zanubrutinib	Not yet
Janus kinase inhibitors	Baracitinib	Not yet
	Ruxolitinib	Not yet
	Tofacitinib	Not yet
Blood-derived products	Convalescent plasma	Not yet
	SARS-CoV-2 immunoglobulins	Not yet
Interleukin-1 inhibitor	Anakinra	Not yet
Interleukin-6 inhibitor	Sarilumab	Not yet
	Tocilizumab	Not yet
	Siltuximab	Not yet
Vaccines	Adenovirus type 5 vector	Not yet
	DNA plasmid vaccine electroporation device	Not yet
	Inactivated	Not yet
	Inactivated + alum	Not yet
	LNP- encapsulated mRNA	Not yet

## Human Alpha-1 Antitrypsin

Human alpha-1 antitrypsin (A1AT) is a 52-kDa glycoprotein that is synthesized in the liver, and is circulated in the blood, and is a natural inhibitor for a set of proteases. Adequate A1AT activity is critical for the prevention of proteolytic tissue damage (Bristow et al., 1998). In individuals with one of several inherited mutations in A1AT, low circulating A1AT levels increase the risk for destructive diseases, particularly emphysema (Chapman et al., 2015). Infusion of plasma purified A1AT protein has proven therapeutic benefits in patients with A1AT deficiency (Stoller and Aboussouan, 2005; Balbi et al., 2016; McElvaney et al., 2017). The pharmacokinetics and safety of A1AT have been well studied. It has been considered safe with infrequent and generally well-tolerated side effects (Petrache et al., 2009). Recent works from our group and others have demonstrated that human A1AT has both anti-inflammatory and anti-SARS-CoV-2 viral effects (Gao et al., 2014; Iskender et al., 2016; Lin et al., 2018; Azouz et al., 2020; Wettstein et al., 2020). This dual role makes it a unique and excellent candidate for COVID-19 treatment.

## The Role of Alpha-1 Antitrypsin in Acute Lung Injury

In addition to its anti-protease and tissue-protective function, A1AT also exerts anti-inflammatory effects, including improving mitochondrial membrane stability, inhibiting apoptosis, inhibiting nuclear factor kappa B (NFκB) activation, modulating pro-vs. anti-inflammatory cytokine balance, and promoting immunologic tolerance. For example, A1AT suppresses tumor necrosis factor-alpha and matrix metalloproteinase-12 production (Churg et al., 2007) and enhances anti-inflammatory cytokine interleukin 10 secretion in macrophages (Janciauskiene, et al., 2007; Ozeri et al., 2012). A1AT inhibits thrombin and plays a role in the regulation of proteases involved in fibrinolysis (Talens et al., 2013). In a graft-versus-host disease murine model, A1AT promoted tolerance in animal models by down-regulating early inflammation and favoring the induction and stabilization of regulatory T cells. A1AT administration promoted the expansion of donor-derived dendritic cells, regulatory T cells, and natural killer cells, and increased survival (Tawara et al., 2012).

A1AT protects the lung from acute injury. A1AT directly inhibited inflammatory responses and caspase three activation, prevented apoptosis in a human lung epithelial cell culture model that simulates preservation and reperfusion process in lung transplantation, and it also protected against ischemia-reperfusion induced acute lung injury in rat lung transplant models (Gao et al., 2014). Moreover, it inhibited coagulation activity, with reduced formation of thrombin-antithrombin complex in plasma, and it reduced inflammatory cytokines and apoptosis in pig lung allografts (Iskender et al., 2016). Furthermore, using a pig-lung transplant survival model, we demonstrated the beneficial effects of A1AT on animal recovery via the lung transplant procedure. We further demonstrated that A1AT protected pig donor lungs during *ex vivo* lung perfusion (EVLP), a technique used to assess marginal donor lungs prior to transplantation (Lin et al., 2018). In severely damaged human lungs declined for clinical transplantation, A1AT significantly improved lung function and reduced vascular leakage and pulmonary edema during EVLP. It showed direct therapeutic benefits to the lung by suppressing multiple cytokines, and inhibiting a potent vasoconstrictor, endothelin 1. Based on these translational research studies, a clinical trial for A1AT in human lung transplantation is in preparation.

## Alpha-1 Antitrypsin as a Biomarker in Coronavirus disease 2019 Patients

Serum interleukin 6 (IL-6) is involved in the cytokine storm seen in COVID-19 patients who developed moderate to severe symptoms (Pedersen and Ho, 2020; Vultaggio et al., 2020). The IL-6/A1AT ratio may reflect the balance between pro- and anti-inflammatory mechanisms. The ratio was markedly higher in COVID-19 patients in the intensive care unit (ICU) than in stable patients; this ratio was further increased in ICU patients with poor outcomes and decreased in cases showing clinical improvement (McElvaney et al., 2020). A1AT has been included as one of the clinical and biological predictors of COVID-19 in two clinical studies (Clinical Trial number: NCT04348396 and NCT04366089) in Italy. Interestingly, serum A1AT levels in SARS patients were significantly lower than those in healthy individuals, and truncated forms of A1AT were significantly higher in sera of SARS patients. The combination of a lower concentration and lower activity of A1AT in SARS patients is likely associated with lung failure and contributes to the development of acute respiratory distress syndrome (Ren et al., 2004). Whether A1AT is also truncated in the serum of COVID-19 patients should be determined.

## Role of Alpha-1 Antitrypsin in Anti-Severe Acute Respiratory Syndrome-Coronavirus-2 Infection

The entry of SARS-CoV2 and other coronaviruses into host cells is through the binding of viral S-protein to angiotensin-converting enzyme 2 (ACE2) located on host cells, which is mediated by host transmembrane protease serine type 2 (TMPRSS2). TMPRSS2-deficient mice showed decreased viral spread in the airways after infection with SARS-CoV (Iwata-Yoshikawa et al., 2019). A1AT inhibits TMPRSS2 proteolytic activity in a dose-dependent manner (Azouz et al., 2020). Wettstein et al. screened a peptide/protein library derived from human bronchoalveolar lavage fluids and identified A1AT as a specific inhibitor of SARS-CoV-2 infection (Wettstein et al., 2020). Moreover, A1AT, as the major human serum protease inhibitor, potently restricts protease-mediated cellular entry of SARS-CoV-2 (Oguntuyo et al., 2020).

## Alpha-1 Antitrypsin as a Drug for Coronavirus disease 2019

**FIGURE 1:**
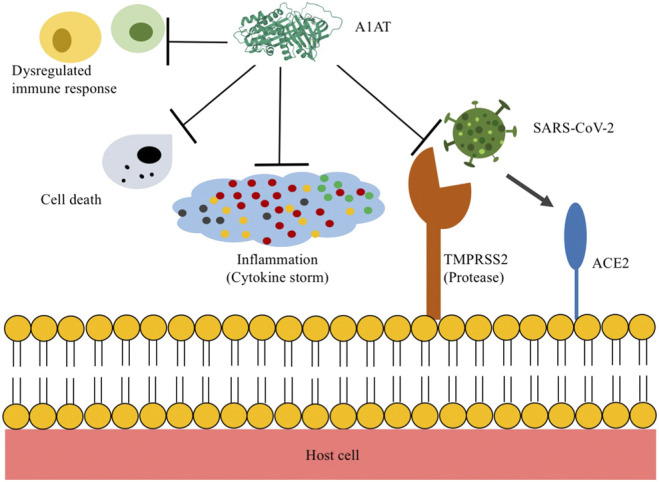
Proposed mechanisms of alpha-1 antitrypsin (A1AT) in COVID-19 treatment. The entry of SARS-CoV2 into host cells is through the binding of viral S-protein to angiotensin converting enzyme 2 (ACE2) located on host cells, which is mediated by the transmembrane serine protease 2 (TMPRSS2). A1AT inhibits TMPRSS2, thus, reduces SARS-CoV-2 infection. In addition, A1AT can reduce acute inflammatory responses, cell death, neutrophil elastase trap formation, coagulative activity, and dysregulated immune responses.

The discovery of anti-SARS-CoV-2 infection, together with the known effects of A1AT on anti-inflammation, anti-cell death, anti-protease, anti-coagulation and immunomodulation, promotes its clinical studies in COVID-19 patients (Figure 1).

Currently, there are three ongoing clinical trials to study A1AT therapy in patients with COVID-19 (Table 2). A phase I randomized placebo-controlled study has been approved on May 13, 2020, in Saudi Arabia. Hospitalized COVID-19 patients will receive A1AT through a nebulizer every 12 hours for 5 days (Clinical Trial number: NCT04385836). A phase II open labelled study has been approved on July 31, 2020, in Spain. Hospitalized patients will receive two intravenous infusion doses of A1AT at 120 mg/kg, on day 1 and day 8 (Clinical Trial number: NCT04495101). Participants in these two trials are patients with moderate COVID-19 only. A similar phase 2 clinical trial was posted on September 10, 2020 to be conducted in the United States of America (Clinical Trial number: NCT04547140). These trials focus on the safety and efficacy of A1AT in preventing the progression of moderate to severe manifestations. It will be important to learn whether intravenous infusion and airway inhalation have similar or different therapeutic efficacy. The success of these trials may lead to further studies using A1AT to treat COVID-19 patients with severe or critical illness. It may also shine light on whether we should give A1AT to patients with only mild symptoms and how to manage patients with A1AT deficiency when they are infected with SARS-CoV-2.

**TABLE 2:** Approved alpha-1 antitrypsin clinical trials for COVID-19 treatment.

Clinical trial	1	2	3
Trial number	NCT04385836	NCT04495101	NCT04547140
Countries	Saudi Arabia	Spain	United States
Randomized prospective	Yes	Yes	Yes
Blinded	Yes (single)	No (open label)	Yes (double)
Phase of clinical trial	I	II	II
Placebo-controlled	Yes	Yes	Yes
Number of participants	150	100	100
Clinical severity	Moderate	Moderate	Moderate
Alpha-1 antitrypsin dosage	8 ml, every 12 h for 5 days	120 mg/kg ×2 doses, day 1 and day 8	120 mg/kg ×2 doses, day 1 and day 8
Control	Usual care plus placebo	Usual care	Usual care plus placebo
Route of administration	Inhalation	Intravenous infusion	Intravenous infusion
Primary measures	Clinical improvement at day 21 according to 7-category ordinal scale	Percentage of participants dying or requiring ICU admission at day 15	Percentage of participants dying or requiring ICU admission at day 15

## Research Agenda

The multiple potential beneficial effects of A1AT makes it a better candidate than antiviral or anti-inflammatory drugs alone for COVID-19 treatment. However, several unanswered questions need to be addressed. First, the anti-SARS-CoV-2 viral infection of A1AT is mainly based on *in vitro* studies. This needs to be validated through *in vivo* studies, with either animal models or clinical samples. SARS-CoV-2 viral titers, TMPRSS2 activities, and levels of ACE2 in serum, bronchoalveolar lavage fluid or tissue biopsy should be examined. These can be further developed as biomarkers for A1AT or other antiviral therapy for clinical prognosis. Serum IL-6 and A1AT levels and their ratio, neutrophil elastase activity, and the formation of NETs should be measured before and during A1AT therapy. Meanwhile, basic and translational research should be conducted to elaborate on the safety, timing, and dosing of A1AT. The potential interactions of A1AT with other drugs should be determined. Moreover, the underlying mechanisms of A1AT on anti-viral, anti-inflammation, anti-cell death, anti-proteases, and anti-coagulation should be studied. Furthermore, Shapira et al. found a significant positive correlation between the combined frequencies of the A1AT deficiency alleles in 67 countries and their reported COVID-19 mortality rates (Shapira et al., 2020). Given the important role that A1AT plays in the prevention and treatment of the pathological process of SARS-CoV-2 infection, patients with A1AT deficiency may be more susceptible to SARS-CoV-2 viral infection with worse clinical outcomes. This should be confirmed through clinical epidemiology studies. These future studies will provide critical information for the clinical application of A1AT in COVID-19 patients.

## Data Availability Statement

The original contributions presented in the study are included in the article/Supplementary Material, further inquiries can be directed to the corresponding author.

## Author Contributions

CY, SK, and ML conceived the design and concepts. CY and ML wrote the manuscript. All authors contributed to the editing and revision of the manuscript and approved the submission.

## Funding

This study was supported by Canadian Institutes of Health Research (PJT-148847) and Government of Ontario, Canada (RE-08-029).

## Conflict of Interest

The authors declare that the research was conducted in the absence of any commercial or financial relationships that could be construed as a potential conflict of interest.
